# Generation of pseudo-CT from a single MRI for PET/MR attenuation correction purposes

**DOI:** 10.1186/2197-7364-1-S1-A74

**Published:** 2014-07-29

**Authors:** Florian Monnier, Hadi Fayad, Julian Bert, Jerome Lapuyade-Lahorgue, Mathieu Hatt, Patrick Veit-Haibach, Gaspar Delso, Dimitris Visvikis

**Affiliations:** INSERM UMR 1101, LaTIM, Brest, France; Nuclear Medicine Department, University Hospital Zurich, Kragujevac, Switzerland

Current MR attenuation correction (AC) approaches suffer from the lack of precision in the detection of bone and the assigned attenuation coefficients. In general, no unique transformation of MR image intensities into attenuation coefficients exists. The purpose of this work is to derive attenuation coefficient maps from a single MR sequence through the generation of a pseudo-CT map using a derived MRI intensity - CT Hounsfield Units (HU) relationship.

**Figure 1:**
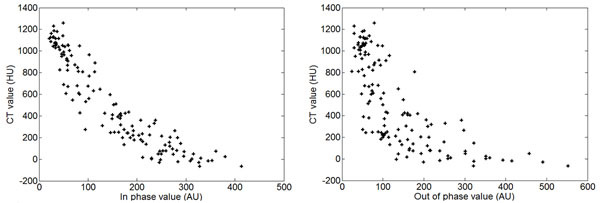
Relations between MRI intensity (in phase and out of phase values) and CT value in ROIs within the pelvic bones.

A retrospective study was undertaken on 10 patients with sequentially performed PET/CT and MR. The LAVA-Flex sequence was investigated, with in-phase (IP) and out-phase (OP) echoes. As a first step, a least square fitting polynomial model (Figure 1) was determined between the CT HU and both

MR images (IP and OP) :

where, a, b, c, d and e are the polynomial model coefficients. To overcome remaining intensity inhomogeneity issues, pseudo-CT soft tissues values were calculated using:

where FF is the voxel-wise fat fraction, HUfat (-102HU) and HUwater (48HU) are reference HU values for fat and water obtained from our dataset.

Pseudo-CT scans were subsequently obtained using the proposed method after segmentation of bones from MRI data using a Fuzzy C-Means algorithm. Pseudo-CT and associated derived attenuations maps were compared with the corresponding acquired CT images. In addition, corresponding PET raw datasets were simulated with lesions placed on key regions of the pelvis.

**Figure 2:**
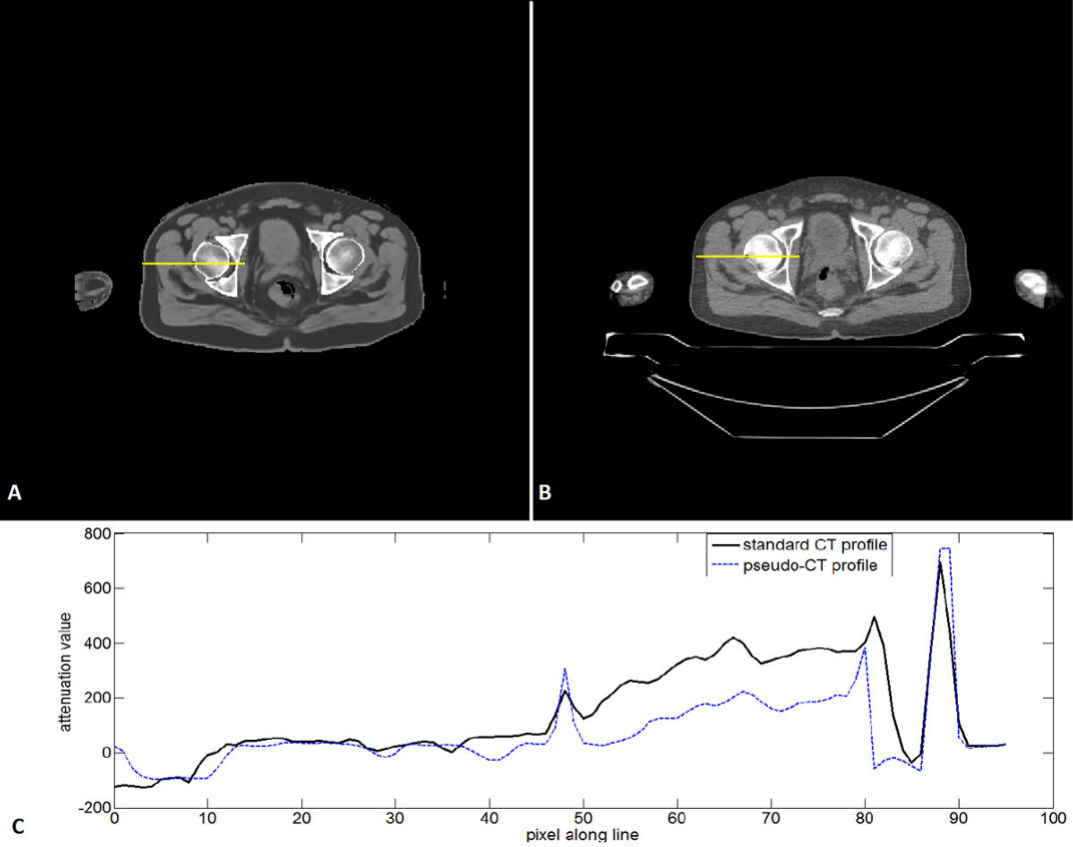
An example of a generated pseudo-CT slice (A) and its corresponding standard CT slice (B). Profiles over the line are plotted underneath (C).

The CT HU - MR intensity generated model gives a mean absolute difference of 92HU (±84HU) in bones, which corresponds to 17.8% (±16.2%) of the mean HU value in bone ROIs. Figure 2 shows a pseudo-CT example as well as its corresponding acquired CT slice with a profile drawn on the bones region. Preliminary results on simulated PET/MR datasets shows errors <8% in the derived SUVs independently of their location in the proximity to bony structures.

